# MicroRNA-451: epithelial-mesenchymal transition inhibitor and prognostic biomarker of hepatocelluar carcinoma

**DOI:** 10.18632/oncotarget.4317

**Published:** 2015-05-27

**Authors:** Jia-Yuan Huang, Kai Zhang, Dong-Qin Chen, Jing Chen, Bing Feng, Haizhu Song, Yitian Chen, Ziman Zhu, Lei Lu, Wei De, Rui Wang, Long-Bang Chen

**Affiliations:** ^1^ Department of Medical Oncology, Jinling Hospital, School of Medicine, Nanjing University, Nanjing, Jiangsu, China; ^2^ Department of Hepatobiliary Surgery, First Hospital Affiliated to the Chinese PLA General Hospital, Beijing, China; ^3^ Liver Disease Center of PLA, The 81th Hospital of PLA, Nanjing, Jiangsu, China; ^4^ Department of Biochemistry and Molecular Biology, Nanjing Medical University, Nanjing, Jiangsu, China

**Keywords:** hepatocellular carcinoma, miR-451, c-Myc, epithelial-mesenchymal transition, invasion

## Abstract

Increasing evidence indicates that dysregulation of microRNAs (miRNAs) plays critical roles in malignant transformation and tumor progression. Previously, we have shown that microRNA-451 (miR-451) inhibits growth, increases chemo- or radiosensitivity and reverses epithelial to mesenchymal transition (EMT) in lung cancer. However, the roles of miR-451 in hepatocelluar carcinoma (HCC) progression and metastasis are still largely unknown. Reduced miR-451 in HCC tissues was observed to be significantly correlated with advanced clinical stage, metastasis and worse disease-free or overall survival. Through gain- and loss-of function experiments, we demonstrated that miR-451 inhibited cell growth, induced G0/G1 arrest and promoted apoptosis in HCC cells. Importantly, miR-451 could inhibit the migration and invasion *in vitro*, as well as *in vivo* metastasis of HCC cells through regulating EMT process. Moreover, the oncogene c-Myc was identified as a direct and functional target of miR-451 in HCC cells. Knockdown of c-Myc phenocopied the effects of miR-451 on EMT and metastasis of HCC cells, whereas overexpression of c-Myc partially attenuated the functions of miR-451 restoration. Furthermore, miR-451 downregulation-induced c-Myc overexpression leads to the activation of Erk1/2 signaling, which induces acquisition of EMT phenotype through regulation of GSK-3β/snail/E-cadherin and the increased expression of MMPs family members in HCC cells. Collectively, these data demonstrated that miR-451 is a novel prognostic biomarker for HCC patients and that function as a potential metastasis inhibitor in HCC cells through activation of the Erk1/2 signaling, at least partially by targeting c-Myc. Thus, targeting miR-451/c-Myc/Erk1/2 axis may be a potential strategy for the treatment of metastatic HCC.

## INTRODUCTION

Hepatocellular carcinoma (HCC) is the fifth common cause of cancer related death worldwide [1]. Although various therapies are used to improve outcomes of HCC patients, such as hepatic resection or liver transplantation, the 5-year survival rate remains only 30% [2]. A major of HCC patients usually cannot have curative surgery which is largely because of distant metastasis and high recurrence ratio at the time of diagnosis [3]. Therefore, a better understanding the molecular mechanisms involved in hepatocellular carcinogenesis contributes to identification of novel prognostic biomarkers and therapeutic targets for HCC.

**Table 1:** Primers of qRT-PCR assay

Name	Primer
**miR-451**	**F:** 5′-ACACTCCAGCTGGGAAACCGTTACCATTA-3′
	**R:** 5′-TGGTGTCGTGGAGTCG-3′
**U6**	**F:** 5′-CTCGCTTCGGCAGCACA-3′
	**R:** 5′-AACGCTTCACGAATTTGCGT-3′
**E-cadherin**	**F:** 5′-CTGAGAACGAGGCTAACG-3′
	**R:** 5′-TTCACATCCAGCACATCC-3′
**N-cadherin**	**F:** 5′-CCACGCCGAGCCCCAGTATC-3′
	**R:** 5′-CCCCCAGTCGTTCAGGTAATCA-3′
**Vimentin**	**F:** 5′-TTGAACGCAAAGTGGAATC-3′
	**R:** 5′-AGGTCAGGCTTGGAAACA-3′
**c-Myc**	**F:** 5′-GGAGGCTATTCTGCCCATTT-3′
	**R:** 5′-CGAGGTCATAGTTCCTGTTGGT-3′
**GAPDH**	**F:** 5′-TGGGTGTGAACCATGAGAAGT-3′
	**R:** 5′-TGAGTCCTTCCACGATACCAA-3′

Epithelial-to-mesenchymal transition (EMT) is considered as a key step of metastatic initiation of tumors, which has been reported to correlate with tumorigenesis, chemoresistance, especially invasion and metastasis [4, 5]. An onset of typical EMT characteristic is come up with mesenchymal markers such as N-cadherin, and Vimentin increased, while epithelial markers decreased simultaneously, which trigger disruption of cell-to-cell adhesion. EMT occurs during HCC progression in response to early metastasis and invasion process, and HCCs with EMT characteristics consistently more venous invasion, metastases and a poor prognosis than those without EMT features [6, 7]. Thus, researches on EMT and its roles in HCC tumorigenesis and metastasis will provide a novel perspective from clinical and translational standpoints.

MicroRNAs (miRNAs) are a class of small noncoding RNAs that block translation or degradation of downstream target messenger RNAs by binding to the 3′ untranslated region (3′-UTR) [8, 9]. Accumulating evidence indicates that miRNA dysfunction is implicated in proliferation, apoptosis, chemoradioresistance, and metastasis of tumors [10-12]. Recently, numerous miRNAs have been reported to be involved with HCC tumorigenesis, such as miR-221, 210, 29c, 100, 520e, 26a and 612 [13-19]. Previously, we have showed that miR-451 up-regulation inhibit growth and induce apoptosis in non-small cell lung cancer (NSCLC) cells. Importantly, restoration of miR-451 could reverse chemo- or radioresistance and EMT phenotypes of lung adenocarcinoma cells [20-22]. Although evidences suggesting miR-451 possibly involved in proliferation and migration of HCC [23, 24], the clinicopathological and prognostic values of miR-451 and its roles in EMT and metastasis of HCC cells remain largely unclear. In this study, we clarified the significance of miR-451 in EMT and metastasis of HCC by using human tissue specimens, *in vitro* assays and animal models. We showed that reduced miR-451 was correlated with higher incidence of metastasis and poor survival of HCC patients. Restoration of miR-451 could reverse EMT and inhibit metastasis of HCC cells *in vitro* and *in vivo*. Furthermore, we testified that miR-451 exerted its anti-metastatic effects by directly targeting the oncogene c-Myc, which led to the activation of Erk1/2 signaling pathway. Our findings highlight the critical roles of miR-451 dysregulation in inhibiting metastasis of HCC through regulation of EMT process.

## RESULTS

### Expression of miR-451 was inversely correlated with metastasis and prognosis in HCC

**Figure 1:**
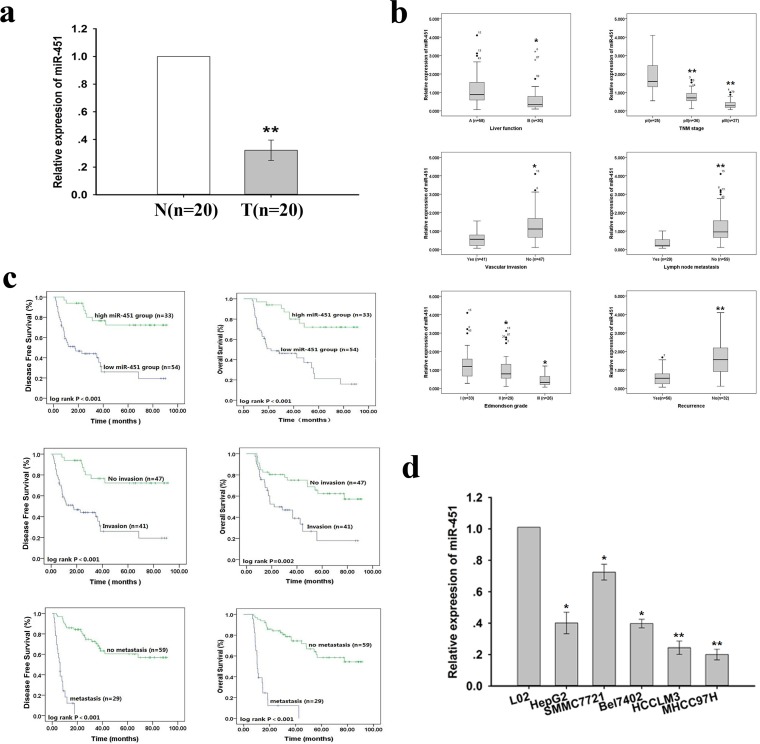
Expression of miR-451 in HCC cell lines and tissue samples **a.** The relative expression level of miR-451 in 20 paired of HCC (T) and the adjacent nontumor tissues (N) was determined by qRT-PCR. The RNU6B small nuclear RNA was used as an internal control and the fold change was calculated by 2^−ΔΔCt^. Cut off value (0.972) was verified by ROC curve. **b.** The lower miR-451 expression in 88 HCC tissue sample was observed to be closely correlated with poorer liver function, advanced TNM stage, higher edmondson grade, higher incidence of vascular invasion or lymph node metastasis and recurrence of HCC patients. **c.** Kaplan-Meier survival plots analysis of the association of miR-451 expression level, vascular invasion and lymph node metastasis with DFS and OS of HCC patients after curative resection. The survival data were compared with the log-rank test. **d.** qRT-PCR detection of relative miR-451 expression level in a normal human hepatocyte cell line (L02) and HCC cell lines (HepG2, SMMC7721, Bel7402, HCCLM3, MHCC97H) with different metastatic potentials. T: HCC tissues; N: the adjacent nontumor tissues. Results represent the average of three independent experiments (mean±SD).*P < 0.05 and **P < 0.01.

To explore the expression and significance of miR-451 in hepatocarcinogenesis, we first detected the expression of miR-451 in 20 paired of HCC and the adjacent nontumor tissues using qRT-PCR. The expression of miR-451 is significantly downregulated in HCC tissues when compared to the adjacent non-tumor tissues, and reduced miR-451 was observed to be significantly associated with advanced TNM stage, lymph node metastasis, vascular invasion and higher Edmondson grade in additional 88 HCC tissues (Figure 1a-1b and Table 3). Furthermore, the Kaplan-Meier survival plots revealed an association of lower miR-451 expression levels with shorter disease-free survival (DFS) and overall survival (OS), and multivariate Cox regression analysis indicated that reduced miR-451 was an independent poor prognostic factor for HCC patients (*P*=0.009; Figure 1c and Table 4). Then, we detected the expression levels of miR-451 in a panel of HCC cell lines with different metastatic potential (Figure 1d), and showed the expression of miR-451 was significantly lower in HCC cells when compared to normal human hepatocyte cell line L02, and the expression level of miR-451 in the highly-metastatic HCC cell lines (HCCLM3 and MHCC97H) was much lower than those in the low-metastatic HCC cell lines (HepG2, SMMC-7721, Bel7402), suggesting that miR-451 downregulation correlates with increased metastatic potential of HCC cells.

**Table 2:** Sequences of miRNAs or shRNAs used in the study

Name	Sequences
**miR-451 inhibitor**	5′-AACUCAGUAAUGGUAACGGUUU-3′
**miR-451 mimics**	**F** 5′-AAACCGUUACCAUUACUGAGUU-3′
	**R** 5′-CUCAGUAAUGGUAACGGUUUUU-3′
**sh-control**	**F** 5′-CCGGGCTTCTCCGAACGTGTCACGTCTCGAGAAGAAACCAGTAAACGTAAGCTTTTTG-3′
	**R** 5′-AATTCAAAAAGCTTCTCCGAACGTGTCACGTCTCGAGAAGAAACCAGTAAACGTAAGC-3′
**sh-c-myc #1**	**F** 5′-CCGGCCCAAGGTAGTTATCCTTAAACTCGAGTTTAAGGATAACTACCTTGggTTTTTG-3′
	**R**5′-AATTCAAAAACCCAAGGTAGTTATCCTTAAACTCGAGTTTAAGGATAACTACCTTGGG-3′
**sh-c-myc #2**	**F** 5′-CCGGCAGTTGAAACACAAACTTGAACTCGAGTTCAAGTTTGTGTTTCAACtgTTTTTG-3′
	**R** 5′-AATTCAAAAACAGTTGAAACACAAACTTGAACTCGAGTTCAAGTTTGTGTTTCAACTG-3′
**sh-c-myc #3**	**F** 5′-CCGGCAGGAACTATGACCTCGACTACTCGAGTAGTCGAGGTCATAGTTCCtgTTTTTG-3′
	**R** 5′-AATTCAAAAACAGGAACTATGACCTCGACTACTCGAGTAGTCGAGGTCATAGTTCCTG-3′

### Restoration of miR-451 significantly suppresses growth, migration and invasion of HCC cells *in vitro*

**Figure 2:**
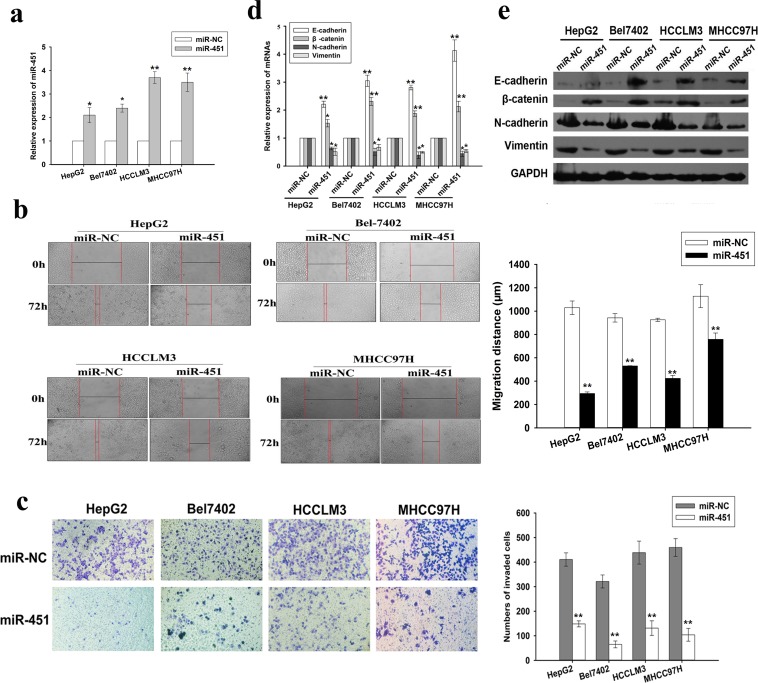
Re-expression of miR-451 negatively regulates HCC cell invasion and metastasis, as well as EMT process *in vitro* **a.** qRT-PCR detection of miR-451 expression in HCC cells stably transfected with pcDNA/miR-451 or pcDNA/miR-NC. The RNU6B small nuclear RNA was used as an internal control. **b.** Wound scratch healing assay of HCC cell migration. A confluent monolayer of HCC cell stably expressing miR-451 or miR-NC was wounded. Photographs were taken immediately (0 h) and at 72 h after wounding, quantification of wound closure was done. **c.** Transwell invasion assay of HCC cells stably expressing miR-451 or miR-NC. Cells in five random fields of view at 100× magnification were counted and expressed as the average number of cells per field of view. **d.** qRT-PCR and **e.** Western blotting detection of mRNA and protein expression of epithelial markers (E-cadherin and β-catenin) and mesenchymal markers (N-cadherin and Vimentin) in HCC cells stably expressing miR-451 or miR-NC, respectively. GAPDH was used as an internal control. Results represent the average of three independent experiments (mean±SD). *P < 0.05 and **P < 0.01.

To further determine the biological significance of miR-451 in HCC, we first performed gain of function experiment by stably transfecting an miR-451-overexpressed plasmid (pcDNA/miR-451) into four human HCC cell lines (Figure 2a). Functional assays indicated that restoration of miR-451 could inhibit growth, induce G_0_/G_1_ arrest and increase apoptosis in HCC cells, which might correlate with a decrease in the cell anti-apoptotic ability (Bcl-2/Bax ratio) and a well know cellular G_1_/S transitional regulator (cyclinD1) (Supplementary Figure 1a-1d). Meanwhile, the effects of miR-451 on migration and invasion of HCC cells were further investigated. Wound scratch healing and matrigel transwell assays indicated that upregulation of miR-451 significantly inhibited migration and invasion of HCC cells (Figure 2b-2c). EMT, the sequence of events that converts adherent epithelial cells into migratory cells, is considered as a key step of metastatic initiation of tumor cells. Thus, we further determined the effects of miR-451 on EMT phenotypes of HCC cells. qRT-PCR and Western blotting assays showed that restoration of miR-451 in HCC cells induced the expression of epithelial markers (E-cadherin and β-catenin) that was accompanied by a concomitant decrease of mesenchymal markers (N-cadherin and Vimentin) (Figure 2d-2e). Likewise, immunofluorecence assay confirmed the expression of those protein markers in pcDNA/miR-451-transfected HCC cells (Supplementary Figure 2a-2b), which demonstrated re-acquisition of epithelial features and a loss of mesenchymal-like features in miR-451-upregulated HCC cells. Therefore, these results indicate that reduced miR-451 plays an important role in promoting HCC tumorigenesis, as well as induction of migration and invasion of HCC cells through regulation of EMT process.

### Restoration of miR-451 expression significantly inhibits *in vivo* tumorigenesis and metastasis of HCC

To confirm the above data *in vivo*, HepG2-miR-451 and Bel7402-miR-451 cells were injected subcutaneously into nude mice. Xenografts tumor volume was measured each week after palpable tumor formed, and mice were sacrificed 8 weeks after tumor implantation. The final tumor volume and weight of HepG2-miR-451 or Bel7402-miR-451 group were significantly smaller than that of HepG2-miR-NC or Bel7402-miR-NC group (Supplementary Figure 3a-3c). Also, qRT-PCR assay confirmed the upregulation of miR-451 and immunostaining assays revealed the increased positive rates of proliferating cell nuclear antigen (PCNA) and ki-67, while TUNEL staining detection of apoptosis showed obvious nuclear fragmentation in HepG2-miR-451 or Bel7402-miR-451 group compared to the control groups (Supplementary Figure 3d-3f). Therefore, restoration of miR-451 could significantly inhibit tumorigenesis of HCC cells *in vivo*.

**Figure 3:**
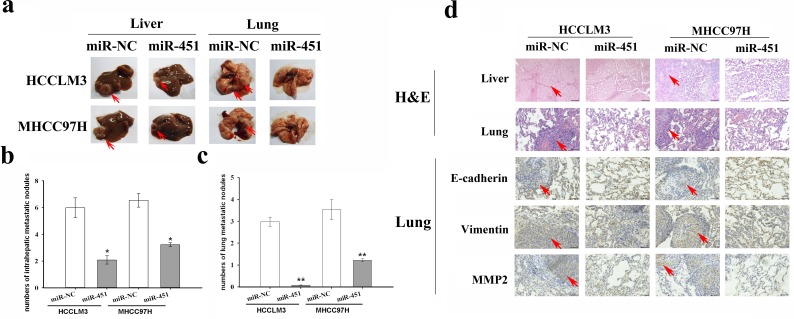
Re-expression of miR-451 inhibits metastasis of HCC cells *in vivo* **a.** Liver primary lesion and lung metastasis as revealed by experimental nude mice (BALB/c nu/nu). Representative photos were shown above, respectively. **b.** The total incidence and number of intrahepatic metastatic nodules in the miR-451-overexpressed groups were much lower than the negative control. **c.** The number of lung metastatic nodules in HCCLM3/miR-451 or MHCC97H/miR-451 group was less than the control groups. **d.** Immunohistochemistry was performed to detect the expression of downstream targets and EMT markers. Vimenin and MMP-2 were dramatically decreased in pcDNA/miR-451 transfected groups, while E-cadherin was enhanced. Results represent the average of three independent experiments (mean±SD). *P < 0.05 and **P < 0.01. Scale bar: 200 μm.

To further determine the effects of miR-451 on the *in vivo* tumor metastasis, the orthotopic HCC models were established using HCCLM3/miR-451 or MHCC97-H/miR-451 cells with a microsyringe. After 8 weeks, mice were sacrificed, and their livers and lungs were isolated, immersed with neutral formalin and prepared for standard histological analysis (Figure 3a). The total incidence and number of lung metastatic nodules, as well as intrahepatic lesions in HCCLM3/miR-451 or MHCC97-H/miR-451 group were much lower than the control groups (Figure 3b-3c). Immunohistochemistry demonstrated the stronger staining of E-cadherin protein and weaker staining of Vimentin and MMP-2 proteins in HCCLM3/miR-451 or MHCC97-H/miR-451 group, when compared to the respective control group (Figure 3d). Collectively, restoration of miR-451 suppresses metastasis of HCC cells *in vivo*.

### c-Myc was identified as a direct and functional target of miR-451 in HCC cells

Previously, we have shown that miR-451 could reverse EMT phenotype of docetaxel-resistant lung adenocarcinoma cells by targeting the oncogene c-Myc. However, whether c-Myc was a functional target of miR-451 in EMT and metastasis of HCC cells needs to be further elucidated. The complementary sequence of miR-451 was exhibited in the 3′-UTR region of c-Myc (1891-1912 nt) (Supplementary Figure 4a), and we previously subcloned the 3′-UTR fragments of c-Myc, in which wild-type and mutant binding sites were harbored immediately downstream of the reporter gene (pLUC-c-Myc/3′-UTR-wt and pLUC-c-Myc/3′-UTR-mut). Luciferase reporter analysis indicated that co-expression of miR-451 significantly reduced the activity of firefly luciferase that carried wild-type but not mutant 3′-UTR of c-Myc, while restoration of miR-451 could downregulate the expression of c-Myc protein in HCC cells and the both xenografts and orthotopic lung implanted model tumors of miR-451-overexpressed HCC cells showed weaker staining of c-Myc protein (Supplementary Figure 4b-4d). These results demonstrate that miR-451 can negatively regulate the expression of c-Myc by directly targeting its 3′-UTR.

### Silencing of c-Myc mimics the effects of miR-451 on phenotypes of HCC cells

**Figure 4:**
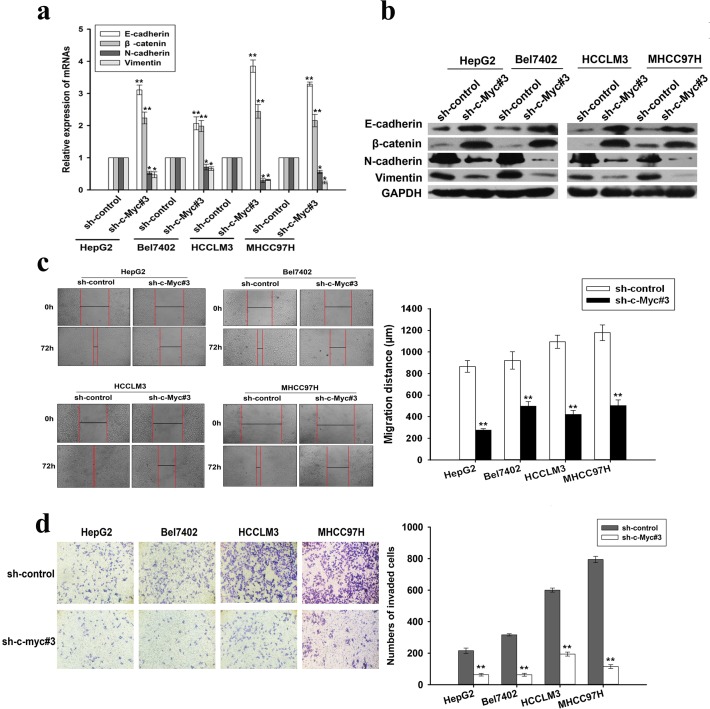
Effects of c-Myc downregulation on EMT, migration and invasion of HCC cells **a.** qRT-PCR and **b.** Western blotting detection of the mRNA and protein expression of epithelial markers (E-cadherin and β-catenin) and mesenchymal markers (N-cadherin and Vimentin) in HCC cells stably expressing sh-c-Myc#3 or sh-control, respectively. GAPDH was used as an internal control. **c.** Wound scratch healing assay of HCC cell migration. A confluent monolayer of HCC cells stably expressing sh-c-Myc#3 or sh-control was wounded. Photographs were taken immediately (0 h) and at 72 h after wounding, quantification of wound closure was done. **d.** Transwell invasion assay of HCC cells stably expressing sh-c-Myc#3 or sh-control. Cells in five random fields of view at 100× magnification were counted and expressed as the average number of cells per field of view. Results represent the average of three independent experiments (mean±SD).*P < 0.05 and **P < 0.01.

To further explore the role of c-Myc in miR-451-mediated EMT and metastasis of HCC cells, we first determine whether knockdown of c-Myc can mimic the effects of miR-451. Three short hairpin RNAs (pSil/shc-Myc #1, 2 or 3) were stably transfected into HCC cells, qRT-PCR and Western blotting confirmed that pSil/shc-Myc3 had the best silencing effect on c-Myc in HCC cells (Supplementary Figure 5a). Then, qRT-PCR and Western blotting assays indicated pSil/shc-Myc#3 could lead to the increased expression of epithelial markers and the decreased expression of mesenchymal markers in HCC cells (Figure 4a-4b). Likewise, immunofluorecence confirmed the changes of EMT-related markers in pSil/shc-Myc#3-transfected HCC cells (Supplementary Figure 6). Furthermore, the capacities of migration and invasion in pSil/shc-Myc#3-transfected HCC cells were significantly reduced in comparison with those in pSil/shcontrol-transfected cells (Figure 4c-4d). Therefore, silencing of c-Myc mimics the effects of miR-451 restoration in HCC cells.

### Restoration of c-Myc reverses the effects of miR-451 on phenotypes of HCC cells

**Figure 5:**
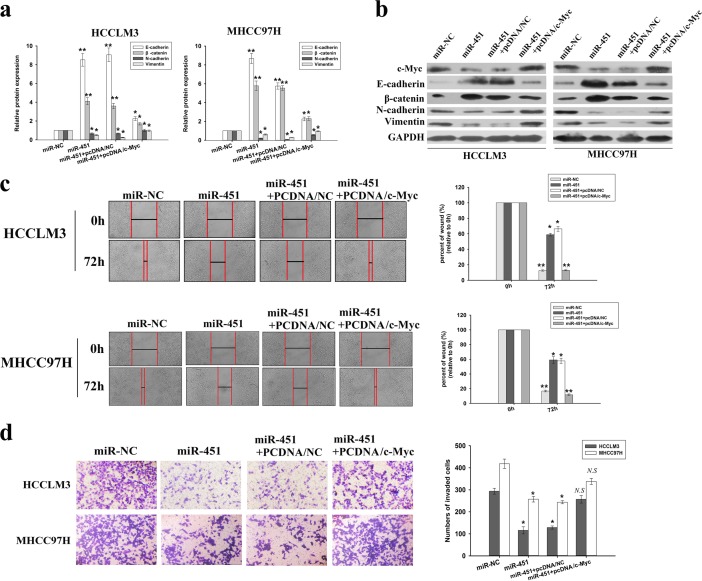
Overexpression of c-Myc reverses the effects of miR-451 upregulation on EMT, migration and invasion of HCC cells **a.** qRT-PCR and **b.** Western blotting detection of the mRNA and protein expression of epithelial markers (E-cadherin and β-catenin) and mesenchymal markers (N-cadherin and Vimentin) in HCC cells stably expressing miR-451 (or miR-NC) or HCC cells stably co-transfected with pcDNA/miR-451 and pcDNA/c-Myc (or pcDNA/NC). GAPDH was used as an internal control. **c.** Wound scratch healing assay of HCC cell migration. A confluent monolayer of HCC cells stably expressing miR-451 (or miR-NC) or HCC cells stably transfected with pcDNA/miR-451 and pcDNA/c-Myc (or pcDNA/NC). **d.** Transwell invasion assay of HCC cells stably expressing miR-451 (or miR-NC) or HCC cells stably co-transfected with pcDNA/miR-451 and pcDNA/c-Myc (or pcDNA/NC). Cells in five random fields of view at 100× magnification were counted and expressed as the average number of cells per field of view. Results represent the average of three independent experiments (mean±SD).*P < 0.05 and **P < 0.01; *N.S*, *P* > 0.05.

We then determined whether restoration of c-Myc could reverse the effects of miR-451 upregulation on EMT and metastasis of HCC cells. The plasmid vector overexpressing c-Myc was stably transfected into HCC cells, and Western blotting confirmed the significant upregulation of c-Myc protein (Supplementary Figure 5b). It was also observed that the co-transfection of pcDNA3/c-Myc and pcDNA/miR-451 could reverse not only the decreased expression of c-Myc and mesenchymal markers but also the increased expression of epithelial markers in HCCLM3 and MHCC97H cells induced by miR-451 upregulation (Figure 5a-5b). Furthermore, the co-transfection could partially reverse the decreased capacities of migration and invasion in HCC cells induced by miR-451 upregulation (Figure 5c-5d). These data indicate that overexpression of c-Myc partially reverse the effects of miR-451 upregulation in HCC cells.

### MiR-451 regulates the expression of EMT-related markers and members of MMPs family through activation of Erk1/2 signaling in HCC through targeting c-Myc

**Figure 6:**
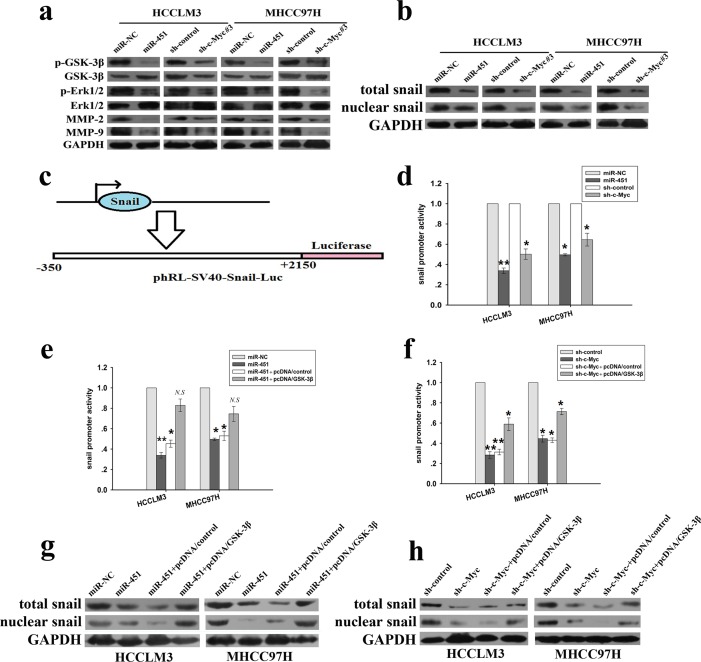
miR-451 regulates the expression of EMT-related markers and members of MMPs family through activation of Erk1/2 signaling in HCC cells by targeting c-Myc **a.** Western blotting detection of the protein expression of total or phosphorylated Erk1/2 (p-Erk1/2), total or phosphorylated GSK-3β (p-GSK-3β), MMP-2 and MMP-9 in HCC cells stably expressing miR-451 (or miR-NC) or HCC cells stably expressing sh-c-Myc#3 (or sh-control), respectively. **b.** Western blotting detection of the protein expression of total or nuclear snail in HCC cells stably expressing miR-451 (or miR-NC) or HCC cells stably expressing sh-c-Myc#3 (or sh-control), respectively. **c.** Schematic model of snail promoter/Luc plasmid. The luciferase reporter containing human snail promoter region 2500 bp from transcription start site were constructed to confirmed the activity of snail promoter. **d.** The histogram of snail promoter activity in HCC cells stably expressing miR-451 (or miR-NC) or sh-c-Myc#3 (or sh-control). Each cell type was transiently transfected with −2.5kb snail promoter/Luc plasmid. Dual-luciferase reporter assays were performed on the lysed cells co-transfected with snail promoter/Luc (firefly luciferase) and phRL-SV (hRenilla luciferase) 24h after co-transfection. Reporter gene activation was determined as a relative ratio of firefly luciferase to hRenilla luciferase activity. **e.** As shown above, the histogram of snail promoter activity in HCC cells stably transfected with pcDNA/miR-451 (or pcDNA/miR-NC) or stably co-transfected with pcDNA/miR-451 and pcDNA/GSK-3β (or pcDNA/control). **f.** As shown above, the histogram of snail promoter activity in HCC cells stably transfected with pSil/sh-c-Myc#3 (or pSil/sh-control) or stably co-transfected with pSil/sh-c-Myc#3 and pcDNA/GSK-3β (or pcDNA/NC). **g.** Western blotting detection of the protein expression of total or nuclear snail in HCC cells stably transfected with pcDNA/miR-451 (or pcDNA/miR-NC) or stably co-transfected with pcDNA/miR-451 and pcDNA/GSK-3β (or pcDNA/NC). **h.** Western blotting detection of the protein expression of total or nuclear snail in HCC cells stably transfected with pSil/sh-c-Myc#3 (or pSil/sh-control) or stably co-transfected with pSil/sh-c-Myc#3 and pcDNA/GSK-3β (or pcDNA/NC). GAPDH was used as an internal control. Results represent the average of three independent experiments (mean±SD).*P < 0.05 and **P < 0.01; *N.S*, *P* > 0.05.

**Figure 7:**
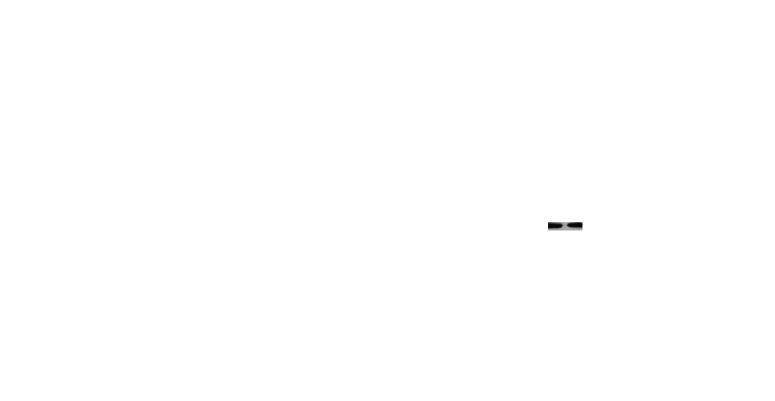
Role of ERK signaling in the effects of miR-451/c-Myc expression on snail or MMPs expression in HCC cells **a.** Western blotting detection of the protein expression of c-Myc, p-Erk1/2 and total Erk1/2 in HCC cells transiently transfected with anti-miR-451 or anti-miR-NC, respectively. **b.** Western blotting detection of the protein expression of total or nuclear snail, total GSK-3β or p-GSK-3β, E-cadherin, β-catenin, N-cadherin, Vimentin, MMP-2 and MMP-9 in anti-miR-451 or pcDNA/c-Myc-transfected HCC cells treated with DMSO or SCH772984 (1.0 μmol/L, a special ERK inhibitor). **c.** The histogram of snail promoter activity in HCC cells transiently transfected with anti-miR-451 (or anti-miR-NC) or stably transfected with pcDNA/c-Myc (or pcDNA/control), respectively. **d.** The histogram of snail promoter activity in anti-miR-451 (or anti-miR-NC)-transfected HCC cells or anti-miR-451-transfected HCC cells treated with DMSO or SCH772984 (1.0 μmol/L). **e.** The histogram of snail promoter activity in pcDNA/c-Myc (or pcDNA/NC)-transfected HCC cells or pcDNA/c-Myc-transfected HCC cells treated with DMSO or SCH772984 (1.0 μmol/L). GAPDH was used as an internal control. Results represent the average of three independent experiments (mean±SD).*P < 0.05 and **P < 0.01; *N.S*, *P* > 0.05.

Previously, overexpression of c-Myc has been reported to induce EMT in mammary epithelial cells via ERK-dependent GSK-3β inactivation and subsequent snail activation and promote tumor cell invasion via activation of MEK-ERK signaling as well as MMPs expression [25, 26]. Therefore, we wondered whether miR-451 participates in the metastasis of HCC through regulation of EMT process by activating ERK signaling. Results of Western blotting showed the decreased expression level of phosphorylated Erk1/2 (p-Erk1/2), with downregulation of MMP-2 and MMP-9 proteins in pcDNA/miR-451 or pSil/shc-Myc#3-transfected HCCLM3 and MHCC97H cells compared to the control cells, but no changes in the expression of total Erk1/2 (Figure 6a). Snail protein levels and its repressor functions are reported to be regulated by GSK-3β and the activity of GSK-3β enzyme is correlated with its Ser-9 phosphorylation level [27]. Here, both restoration of miR-451 and c-Myc downregulation could induce the decreased expression of p-GSK-3β (Ser-9) protein, but no changes of total GSK-3β protein (Figure 6a). Snail, a zinc finger family of transcriptional repressors, functions as a potent repressor of E-cadherin, and GSK-3β inhibition induces stability and increased nuclear levels of snail protein [28]. Thus, we will analyze whether miR-451/c-Myc affects the expression of snail protein and activity of its promoter. It was observed that both miR-451 upregulation and c-Myc downregulation induced the decreased expression of total and nuclear snail protein (Figure 6b). The luciferase reporter containing human snail promoter regions (2500 bp) from transcription start site were previously constructed to confirm the activity of snail promoter, and luciferase reporter analysis indicated that the transcriptional activity of snail promoter in both miR-451-upregulating and c-Myc-downregulating HCC cells was significantly inhibited (Figure 6c-6d). To further determine the roles of GSK-3β on the activity of snail promoter and the expression of snail protein, pcDNA/GSK-3β vector was previously constructed and the co-transfection of pcDNA/GSK-3β with pcDNA/miR-451 or pSil/shc-Myc#3 could partially restore the decreased activity of snail promoter and expression of total or nuclear snail protein in HCC cells induced by miR-451 upregulation or c-Myc downregulation (Figure 6e-6f). At the same time, transfection of anti-miR-451 could induce the increased expression of c-Myc and p-Erk1/2 proteins in HCCLM3 and MHCC97H cells (Figure 7a-7b). Also, miR-451 downregulation could lead to the decreased expression of epithelial markers and the increased expression of mesenchymal markers, total and nuclear Snail, p-GSK-3β, MMP-2 and MMP-9 proteins in above HCC cells, while both miR-451 downregulation and c-Myc overexpression could lead to the increased activity of snail promoter (Figure 7c-7d). Importantly, the increased activity of snail promoter in HCCLM3 cells induced by miR-451 downregulation or c-Myc overexpression could be both partially reversed by SCH772984 (a special ERK inhibitor, 1.0 μmol/L) (Figure 7e-7f). Meanwhile, the increased expression of those proteins (total and nuclear snail, p-GSK-3β, N-cadherin and Vimentin, MMP-2 and MMP-9) and the decreased expression of E-cadherin and β-catenin in anti-miR-451 or pcDNA/c-Myc-transfected HCCLM3 cells could be also partially reversed by SCH772984 (Figure 7c). These results clearly demonstrate that activation of Erk1/2 signaling mediates miR-451/c-Myc-induced EMT and metastasis in HCC cells by regulating the expression of EMT-related markers and members of MMPs family.

### Upregulation of c-Myc in HCC tissues, inversely correlated with miR-451 expression, correlates with metastasis and poor survival of patients

**Figure 8:**
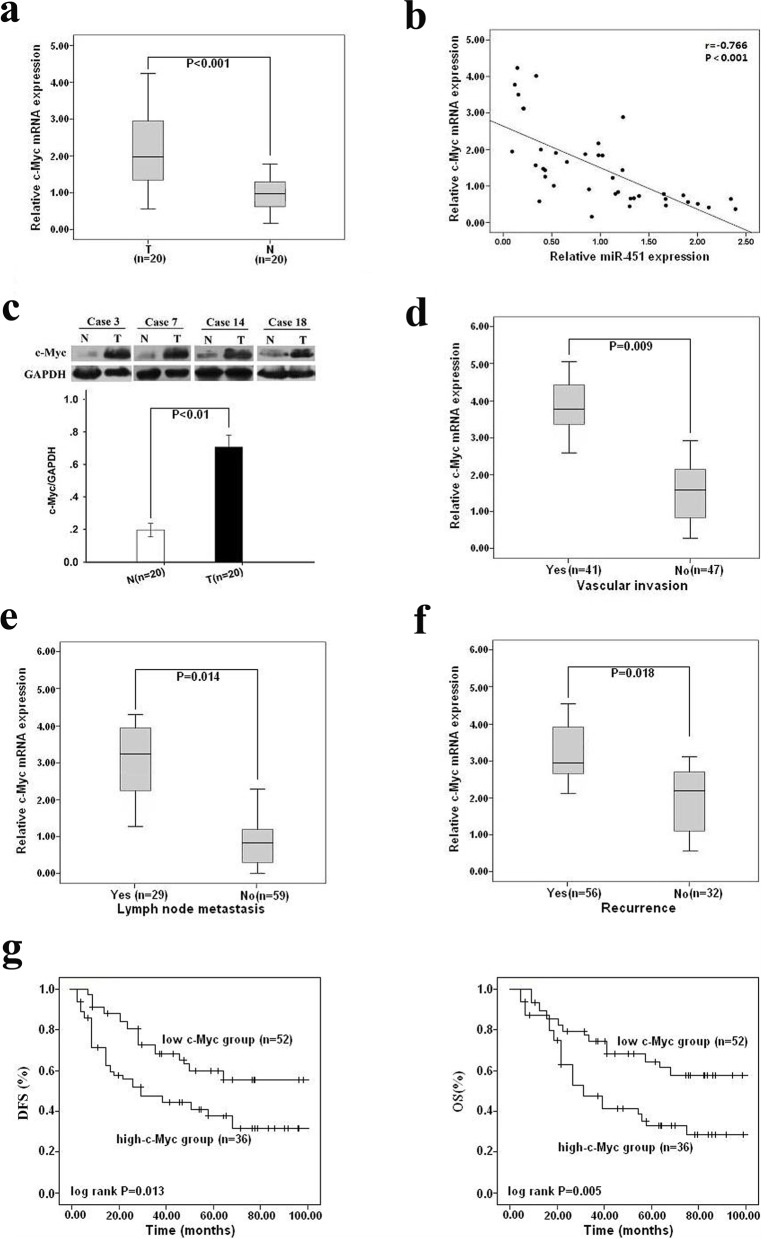
Upregulation of c-Myc in HCC tissues, inversely correlated with miR-451 expression, is associated with metastasis and poor survival of patients **a.** qRT-PCR detection of relative c-Myc mRNA expression in 20 paired of HCC tissues (T) and the adjacent nontumor tissues (N) (*P* < 0.001). **b.** Statistically significant inverse correlation between miR-451 and c-Myc mRNA expression in above tissues (n = 40; r = −0.766, *P* < 0.001). **c.** Western blotting detection of relative c-Myc protein expression in above tissues (*P* < 0.01). **d.** qRT-PCR detection of relative c-Myc mRNA expression in HCC tissues with or without vascular invasion (*P* = 0.009). **e.** qRT-PCR detection of relative c-Myc mRNA expression in HCC tissues with or without lymph node metastasis (*P* = 0.014). **f.** qRT-PCR detection of relative c-Myc mRNA expression in HCC tissues with or without tumor recurrence (*P* = 0.018). **g.** The median value of c-Myc mRNA in all HCC tissues (n = 88) was 2.08 and used as a cutoff value, and all patients were divided into two groups: high-c-Myc group (≥2.18; n = 36) and low-c-Myc group (< 2.18; n = 52). Kaplan-Meier survival plots analysis of the association of c-Myc mRNA expression level with DFS (*P* = 0.013) and OS (*P* = 0.005) of HCC patients after curative resection. The survival data were compared with the log-rank test. GAPDH was used as an internal control. Results represent the average of three independent experiments (mean±SD).

Next, we performed qRT-PCR to detect the expression of c-Myc mRNA in above 20 paired HCC and the adjacent nontumor tissues, and showed that the relative expression level of c-Myc mRNA in HCC tissues was significantly higher than that in the matched adjacent nontumor tissues (Figure 8a), and a significant inverse correlation between the expression level of miR-451 and that of c-Myc mRNA was observed in the 20 paired HCC tissues (r = −0.776; *P*<0.001; Figure 8b). Likewise, Western blotting confirmed the high expression level of c-Myc protein in HCC tissues compared to the adjacent nontumor tissues (Figure 8c). Meanwhile, high c-Myc mRNA expression in HCC tissues was observed to significantly correlate with higher incidence of vascular invasion, lymph node metastasis and recurrence of patients (Figure 8d-8f). Furthermore, patients with high c-Myc expression showed much lower PFS or OS than those with low c-Myc expression (*P*=0.013 or 0.005; Figure 8g). These results demonstrated that upregulation of c-Myc in HCC tissues inversely correlated with miR-451 expression and significantly correlated with metastasis and poor prognosis of patients.

## DISCUSSION

Currently, more and more miRNAs were also converged to maintain distinctive characters of various processes, including tumor initiation, development as well as metastasis [29]. Previously, we have reported that miR-451 inhibits growth and promotes apoptosis in NSCLC cells partially by targeting RAB14 [20]. Furthermore, we showed that miR-451 could reverse chemo- or radioresistance and EMT phenotypes of docetaxel-resistant lung adenocarcinoma cells by targeting c-Myc [21, 22]. Also, miR-451 is reported to be downregulated in many other human cancers, suggesting that miR-451 functions as a tumor suppressor [30]. Recently, the correlations of miR-451 expression with HCC are also reported. Li’ et al firstly reported that miR-451 inhibits cell proliferation in HCC through direct suppression of IKK-β [23]. Also, Lv’ et al reported that miR-451 regulates activating transcription factor 2 expression and inhibits HCC cell migration [24]. However, the clinicopathological and prognostic significance of miR-451 in HCC remains largely unclear. In this study, qRT-PCR testified that miR-451 was frequently downregulated in both HCC tissues and liver cancer cell lines. Interestingly, the expression of miR-451 in high-metastatic HCC cell lines was significantly lower than that in low-metastatic HCC cell lines. Also, reduced miR-451 expression in HCC tissues was observed to be closely with advanced TNM stage, lymph node metastasis, vascular invasion, edmondson grade and poor survival of patients, and multivariate analysis indicated that low miR-451 expression was an independent poor prognostic factor for patients. These data demonstrated that reduced miR-451 might play critical roles in HCC progression.

To further demonstrate the effects of miR-451 expression on malignant phenotypes of HCC cells, we then performed gain- and loss-of function experiments. It was observed that restoration of miR-451 could significantly inhibit *in vitro* growth and *in vivo* tumorigenesis of HCC cells by inducing G_0_/G_1_ arrest and apoptosis enhancement, which were consistent with the report by Li’ et al [23]. Likewise, another report reveals a prominent role of miR-451 as a tumor suppressor regulating HCC growth in a caspase-3-dependent manner [31]. Previously, we have shown that miR-451 significantly suppresses growth in NSCLC, and the tumor-suppressive effects of miR-451 are reported in other human cancers. For example, miR-451 was reported to inhibit growth of colorectal carcinoma and glioma cells via targeting PI3k/Akt pathway [32, 33]. Liu’ et al showed that transfection of miR-451 mimics not only inhibited growth and invasion of tumor cells but also decreased angiogenic ability of HUVEC cell by targeting the IL6R pathway [34]. Interestingly, it was also reported that microRNA-451 regulates LKB1/AMPK signaling and allows adaptation to metabolic stress in glioma cells [35]. Under condition of glucose withdrawal, miR-451 downregulation increased cell survival and migration, suggesting that miR-451 might be a conditional switch controlling glioma cell proliferation and migration [36]. Our and other reports showed that upregulation of miR-451 could reverse the resistance of lung adenocarcinoma cells to cisplatin and docetaxel [21, 37]. Additionally, miR-451 was reported to be involved in resistance of the MCF-7 breast cancer cells to chemotherapeutic drug doxorubicin, while tamoxifen downregulation of miR-451 increases 14-3-3ζ and promotes breast cancer cell survival and endocrine resistance [38, 39]. Lopotová and his colleagues testified the more complex relationship of miR-451 and BCR-ABL, suggesting that miR-451 might be a putative predictor marker of Imatinib therapy response in chronic myeloid leukemia [40]. Collectively, these results clearly demonstrated that miR-451 functions as a tumor suppressor in human cancers.

EMT, the key process that drives metastasis, is characterized by loss of the epithelial marker, increased expression of the mesenchymal marker, and enhanced migratory and invasive behaviors [41]. Recently, the involvement of miRNAs in regulation of the EMT process of tumor cells was increasingly demonstrated. For example, ECM proteins and peptidases have been identified to be directly regulated by miR-200 family, which could alter the tumor microenvironment to restrain the EMT process in lung cancer [42]. In another study, EMT mediated by the transcriptional repressor snail1 expression was regulated by miR-34 when wild-type p53 loss of function or mutation in multiple tumor cells [43]. Presently, only a few miRNAs are reported to be involved in regulation of EMT process in HCC cells. Zhang’ et al showed that miR-148a suppresses EMT of hepatoma cells by targeting Met/Snail signaling [44]. Also, miR-491 is reported to be involved in metastasis of HCC by inhibitions of matrix metalloproteinase and EMT [45]. Conversely, other miRNAs promoting EMT of HCC cells are also found. For example, Over-expression of miR-106b could promote cell migration and metastasis in HCC by activating EMT process [46]. MiR-216a/217-induced EMT targets PTEN and SMAD7 could promote drug resistance and recurrence of liver cancer [47]. However, the miRNA networks in EMT process of HCC remains largely unknown. Our previous study has shown that re-expression of miR-451 reverses EMT phenotype and inhibits metastasis of docetaxel-resistant lung cancer cells, but whether miR-451 plays important roles in formation of EMT phenotype in HCC cells is unknown. By qRT-PCR and Western blotting assays, we showed that re-expression of miR-451 could significantly downregulate the expression of mesenchymal markers (E-cadherin and β-catenin) and increase the expression of epithelial markers (N-cadherin and Vimentin) at both mRNA and protein levels, which were also confirmed by immunofluorecence assay. Furthermore, we showed that restoration of miR-451 could reduce the capacities of *in vitro* migration and invasion of HCC cells. More importantly, the *in vivo* metastasis of HCC cells was also inhibited by miR-451 upregulation. These above results demonstrated that restoration of miR-451 could reverse EMT phenotypes and suppress metastasis of HCC cells.

The important roles of miRNAs are to regulate the expression of their downstream specific targets through mRNA cleavage and / or by inhibition of translation. One miRNA could hold multiple target genes, effect gene could be repressed by multiple miRNAs [48]. Thus, to further explore the molecular mechanisms by which miR-451 exerts its anti-metastatic functions, identification of its functional targets is essential. In our previous study, the pro-oncogene c-Myc was identified as a direct and functional target of miR-451 in lung cancer. c-Myc is a nuclear transcriptional factor and most of its targeting genes are crucial for ribosome biogenesis and protein synthesis, which are indispensable for cell proliferation and development [49]. It has been reported that c-Myc is highly expressed in many human cancers, including HCC [50]. Likewise, in T cell acute lymphoblastic leukemia (T-ALL), repression of tumor suppressor miR-451 is essential for NOTCH1-induced oncogenesis by directly targeting c-Myc [51]. In HCC, Li’ et al showed that miR-451 upregulation led to downregulation of cyclin D1 and c-Myc through inhibition of NF-κB pathway initiated by direct targeting of the IKBKB 3′-untranslated region [21], but it is still unclear whether miR-451 can directly regulate the expression of c-Myc through binding to the promoter region of c-Myc. To testify this, we first performed luciferase reporter assay, and showed that the luciferase activity could be reduced by miR-451. In addition, re-expression of miR-451 could downregulate the expression of c-Myc protein in HCC cells. More importantly, silencing of c-Myc could mimic the effects of miR-451 upregulation on malignant phenotypes of HCC cells, while overexpression of c-Myc could partially reverse the phenotypical changes of HCC cells induced by miR-451 upregulation. Furthermore, the expression of c-Myc in HCC tissues was significantly higher than that in the adjacent nontumor tissues, and statistically significant inverse association between miR-451 and c-Myc expression in HCC tissues was observed. These data clearly demonstrated that c-Myc was a direct and functional target of miR-451 in HCC. Subsequently, we further investigated the possible downstream signaling pathways. Although the precise role of c-Myc in metastasis process and EMT is still elusive, multiple studies have shown that c-Myc controls and supports this complex multistep process at different stages of human cancers [52]. Here, we showed that both miR-451 upregulation and c-Myc downregulation induced the decreased expression of p-Erk1/2, p-GSK-3β, MMP-2 and MMP-9 proteins, the decreased expression of total or nuclear snail proteins, and eventually the reduced activity of snail promoter. Importantly, overexpression of GSK-3β could partially reverse the decreased activity of snail promoter reporter induced by miR-451 upregulation or c-Myc downregulation, while the ERK inhibitor could not only reverse the increased expression of p-GSK-3β, total or nuclear snail, E-cadherin and MMPs in HCC cells induced by miR-451 downregulation, but also reverse the increased activity of snail promoter induced by miR-451 downregulation and c-Myc overexpression. These results clearly demonstrated that miR-451/c-Myc/Erk1/2/GSK-3β or MMPs signaling pathway might be involved in EMT and metastasis of HCC cells (Supplementary Figure 7).

In summary, we identified that reduced miR-451 correlates with early recurrence, poor survival and metastasis of HCC patients. Re-expression of miR-451 reduces tumorigenesis, reverses EMT and inhibits metastasis in HCC cells via inactivation of the Erk1/2 signaling pathway, at least partially by targeting c-Myc. Therefore, miR-451 may be a potential prognostic biomarker, and targeting this novel miR-451/c-Myc/Erk axis will be a promosing strategy for the treatment of metastatic HCCs.

## MATERIALS AND METHODS

### Patients and tissue samples

20 paired primary HCC and corresponding nontumor liver tissues, as well as 88 additional primary HCC surgical specimen were collected at the Department of Hepatobiliary Surgery of First Hospital Affiliated to the Chinese PLA General Hospital and the Liver Disease Center of the 81th Hospital of PLA from 2005 to 2007. The informed consent was signed from all the patients. None of preoperative treatment was done. International Union Against Cancer (UICC) TNM classification were used to verify the clinical stage. All clinical records were reviewed, and details were listed in Table 3. The pathological judgment was determined by two senior pathologists with double-blind. All the cases were closely followed up for recurrence or death. The tissues were rapidly frozen and stored in liquid nitrogen. Scientific and ethic approval was certificated by the Ethics Committee of Jiangsu Province Medical Association.

**Table 3:** Correlations between miR-451 expression and clinicopathological factors of HCC patients

	miR-451 expression			
Factors	n	low (n=55)	high (n=33)	*P*-value
Gender				0.676
Male	61	39	22	
Female	27	16	11	
Age (years)				0.759
>55	49	36	20	
≤55	39	19	13	
Family history				0.526
Yes	30	18	13	
No	58	37	20	
Alcohol intake				0.648
No	23	36	9	
Yes	65	19	29	
HBV infection				0.310
Yes	61	36	25	
No	27	19	8	
Tumor diameter (cm)				0.491
≤5.0	65	42	23	
>5.0	23	13	10	
Liver function				0.429
Child-Pugh A	54	32	22	
Child-Pugh A	34	23	11	
TNM stage				0.003*
I	19	9	10	
II	36	13	23	
III	34	26	8	
Lymph node metastasis				0.022*
Yes	32	25	7	
No	56	30	26	
Vascular invasion				0.021*
Yes	46	34	12	
No	42	21	21	
Edmondson grade				0.038*
I	29	13	16	
II	29	19	10	
III	30	23	7	

Abbreviation: HCC: hepatocellular carcinoma; HBV: hepatitis B virus

* *P*<0.05.

**Table 4:** Univariate and multivariate analysis of prognostic variables by Cox regression analysis

Clinicopathological factors	Univariate analysis		Multivariate analysis	
	RR (95% CI)	*P*-value	RR (95% CI)	*P*-value
TNM stage (I/II/III)	1.122(0.54-2.34)	0.001*	34.1 (8.51-136.7)	0.001*
Edmondson grade (I/II/III)	1.198(0.98-4.04)	0.02*	2.64 (1.39-5.02)	0.003*
miR-451 expression (low / high)	1.52 (1.08-2.12)	0.016*	2.09 (1.20-3.63)	0.009*

RR: relative ratio; 95% CI: 95% confidence interval.

### Cell culture

Four human hepatoma cell lines (HepG2, Bel7402, HCCLM3, MHCC97H) and a normal human hepatocyte cell line (L02) were purchased from the Cell Bank of Shanghai institute of cell biology, Chinese Academy of Medical Sciences (Shanghai, China). All these cell lines were cultured in Dulbecco's modified Eagle's medium (DMEM) supplemented with 10% fetal bovine serum (GIBCO), 100U/ml penicillin and 100ug/ml streptomycin at 37°C in a humidified atmosphere of 5% CO_2_ and 95% air.

### Cell transfection

The miR-451-overexpressing plasmid vector (pcDNA/miR-451) and GSK-3β-overexpressing plasmid vector (pcDNA/GSK-3β) were previously constructed and preserved in our lab. miR-451 inhibitor (anti-miR-451) and mimics, as well as c-Myc short hairpin RNA (pSil/sh-c-Myc#1, 2, 3), and nonspecific control shRNA (pSil/control) were all chemically synthesized by Genechem (Shanghai, China), as listed in Table. 2. The stable transfection was performed using Lipofectamine 2000 (Invitrogen, Carlsbad, CA) according to the manufacturer's protocol as described previously [20].

### Quantitative real-time PCR (qRT-PCR) assay

Real-time qRT-PCR analysis of mature miRNA and mRNA was performed using TaqMan^®^ MicroRNA Cells-to-CT™ Kit as described previously [53, 54].

### Western blotting assay

The expression of E-cadherin, N-cadherin, Vimentin, β-catenin-c-Myc, cyclinD1, Bax, Bcl-2, NF-κB, NF-κB/65, snail, GSK-3β, p-GSK-3β, Erk1/2, p-Erk1/2, MMP-2, MMP-9 and GAPDH proteins was detected using Western blotting assay as previously described [21]. All bodies were purchased from Univ-bio Inc (Shanghai, China).

### Colony formation assay

The cells were transplanted in a 6-well culture dish and maintained in DMEM containing 10% FBS. The medium was renewed 24 hours later. After 2 weeks, cells were stained with 0.1% crystal violet after fixed with methanol. All the visible colonies were calculated manually.

### Flow cytometric analysis

An annexin V-fluorescein isothiocyanate (FITC) apoptosis detection Kit and cell cycle detection Kit (KeyGEN Biotech) was used according to the manufacturer's instructions as previously described [20].

### *In vitro* migration and invasion assays

The wound healing and transwell assays were performed as described previously [55].

### Immunofluorescence assay

The expression E-cadherin, β-catenin, N-cadherin and Vimentin proteins in HCC cells was determined by an immunofluorescence assay as described previously [22].

### Immunohistochemistry assay

Paraffin-embedded, formalin-fixed tissues were immunostained for E-cadherin, Vimentin, MMP-2, c-Myc, Ki-67, PCNA proteins as previously described [20].

### *In vivo* tumor growth and metastasis assays

Animal experiments were certificated by the Institutional Committee for Animal Research. Female athymic BALB/c nude mice (6 week old) were purchased from the Department of comparative medicine (Jinling Hospital, Nanjing, China). Subcutaneous xenografts or orthotopic metastatic model were establilshed as as previously described [55].

### Luciferase reporter assay

Luciferase reporter containing wild type 3′-UTR of c-Myc (pLUC/c-Myc/3′-UTR-wt) and mutant reporter (pLUC/c-Myc/3′-UTR-mut) were previously established and preserved in our lab. The luciferase assay was performed as previously described [21, 22].

### Promoter reported gene analysis

The −2.5kb human snail promoter was cloned and insert in phRL-SV luciferase reporter vector which was purchased from Promega. Cor. (Madison, WI, USA). The promoter activity was detected by Dual-Luciferase Reporter Assay kit (Promega, USA) as previously described [21, 22].

### Statistical analysis

All statistical analyses were performed with SPSS version 17.0 for Windows (SPSS Inc., Chicago, IL). Qualitative data were determined by descriptive statistics, including chi-square test or Fisher's exact test when appropriate. Survival analysis was estimated by Kaplan-Meier method. Cox proportional hazards regression was applied for multivariate analysis. *P*<0.05 was considered statistically significant.

## SUPPLEMENTARY MATERIAL FIGURES


