# Supplementary material for: MicroRNA-451: epithelial-mesenchymal transition inhibitor and prognostic biomarker of hepatocelluar carcinoma
Source: Oncotarget. 2015 May 27;6(21):18613–30. doi: 10.18632/oncotarget.4317 (PMC4621914; doi:10.18632/oncotarget.4317)
Supplement: Supplementary file 1 [file oncotarget-06-18613-s001.pdf]

MicroRNA-451: epithelial-mesenchymal transition inhibitor and prognostic biomarker of hepatocelluar carcinoma

Supplementary Material

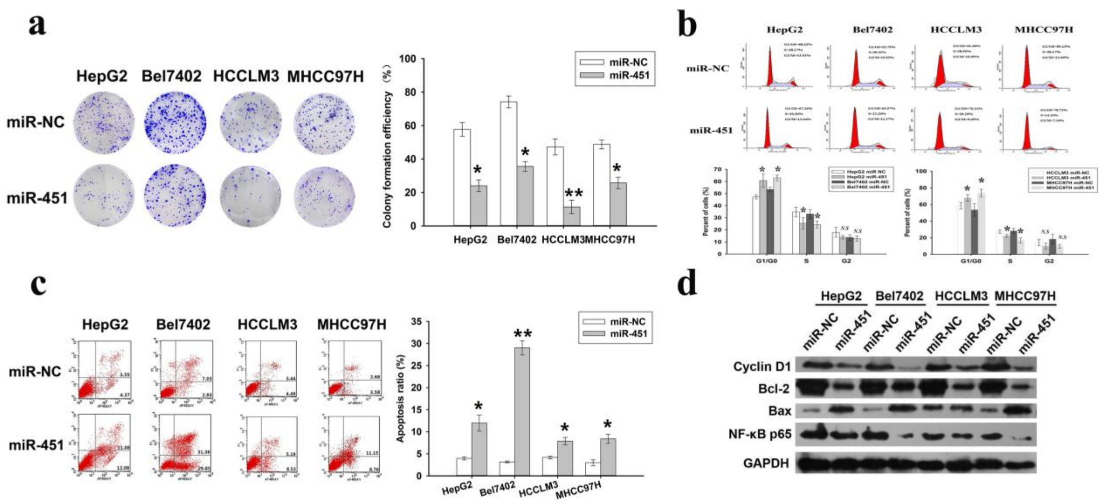

Supplementary Figure 1: Effects of miR-451 expression on growth, cell cycle and apoptosis of HCC cells. (a) Representative results of colony formation in HCC cells stably expressing miR-451 or miR-NC. (b) Flow cytometry detection of cell cycle in HCC cells stably expressing miR-451 or miR-NC. (c) Flow cytometry detection of apoptosis in HCC cells stably expressing miR-451 or miR-NC. (d) Western blotting detection of the protein expression of cyclinD1, Bcl-2, Bax and NF-κB/p65 in HCC cells stably expressing miR-451 or miR-NC. GAPDH was used as an internal control. Results represent the average of three independent experiments (mean±SD). *P<0.05 and **P<0.01; *N.S*, P>0.05.

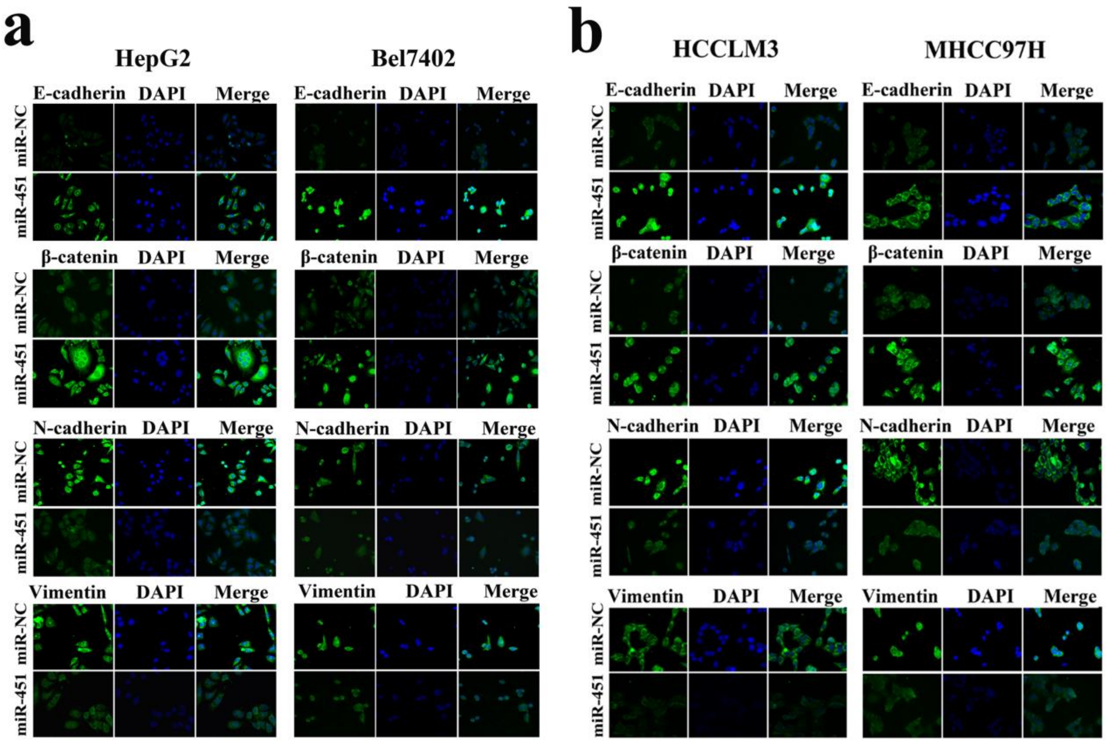

Supplementary Figure 2: Immunofluorecence assay detection of EMT-related protein markers in HCC cells stably expressing miR-451 or miR-NC. (a) Immunofluorescence analysis of epithelial protein markers (E-cadherin and β-catenin) and mesenchymal protein markers (N-cadherin and Vimentin) in low-metastatic HCC cells (HepG2 and Bel7402) stably expressing miR-451 or miR-NC. (b) Immunofluorescence analysis of epithelial protein markers (E-cadherin and β-catenin) and mesenchymal protein markers (N-cadherin and Vimentin) in low-metastatic HCC cells (HCCLM3 and MHCC97H) stably expressing miR-451 or miR-NC. Merged pictures represent overlays of protein markers (green) and nuclear staining by 4',6-diamidino-2-phenylindole (DAPI; blue). Scale bar: 200 μm.

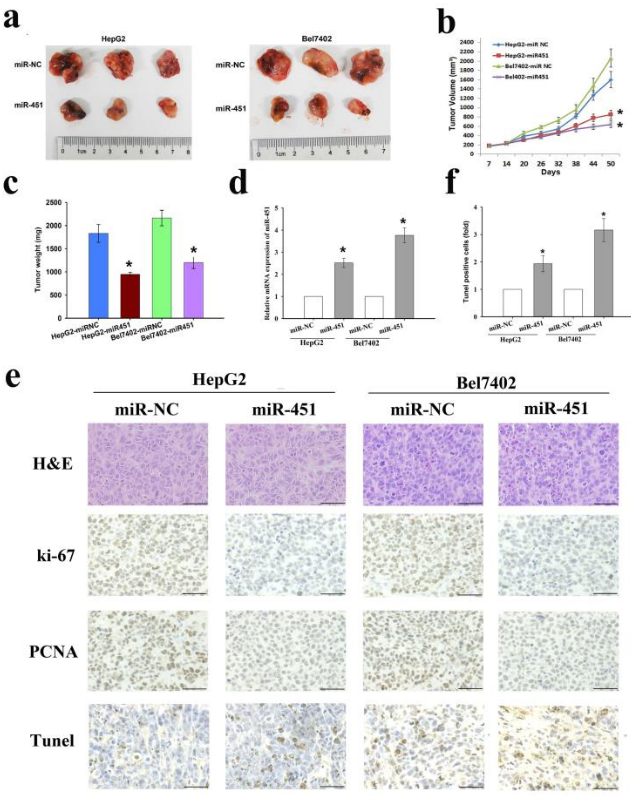

Supplementary Figure 3: Overexpressed miR-451 suppressed HCC proliferation capacity *in vivo*. HepG2 and Bel7402 cells stably expressing miR-451 or control vector were injected subcutaneously into nude mice for animal models. (a) Representative photographs of Xenograft tumors formed in 8 weeks after the subcutaneous transplantation were exhibited. MiR-451 overexpressed group was extremely smaller than control ones. (b) Xenografts tumor volume was measured each week after palpable tumor formed. The final tumor volume of HepG2 cells stably transfected with miR-451 was 851.17 mm 3, which was significantly smaller than tumors in the control group (1602.50 mm 3; P<0.05). Similar results were also found in Bel7402, which stable transfected with miR-451 group showed weaker proliferation ability than the control group (the final tumor volume were 643.33 mm 3 vs 2064.34 mm 3). (c) the tumor weight of HepG2 transfected with miR-451 was much smaller than control vector (948.32mg vs 1830.52mg), the tumor weight of Bel7402 was consistent with these results (1203.76mg vs 2163.49mg). (d) Expression of miR-451 in Xenograft tumors were confirmed by q-PCR. miR-451 was upregulated by stable transfection. (e) Hematoxylin and eosin (H & E)-stained and relative markers Immunostaning in transplanted tumors. The positive rate of proliferating cell nuclear antigen (PCNA) and ki-67 in pcDNA/miR-451 transfected HCC cells were decreased in comparison with control group. Tunnel staining revealed obvious nuclear fragmentation in pcDNA/miR-451-transfected HCC cells, in contrast to control ones. Photograph were taken under magnification: 200×. *P<0.05, versus nonspecific control (NC). (f) Apoptosis ratio was measured by the histogram of Tunel positive cells which was normalized by mean±SD in three independent experiments. Each experiment was repeated in triplicate.

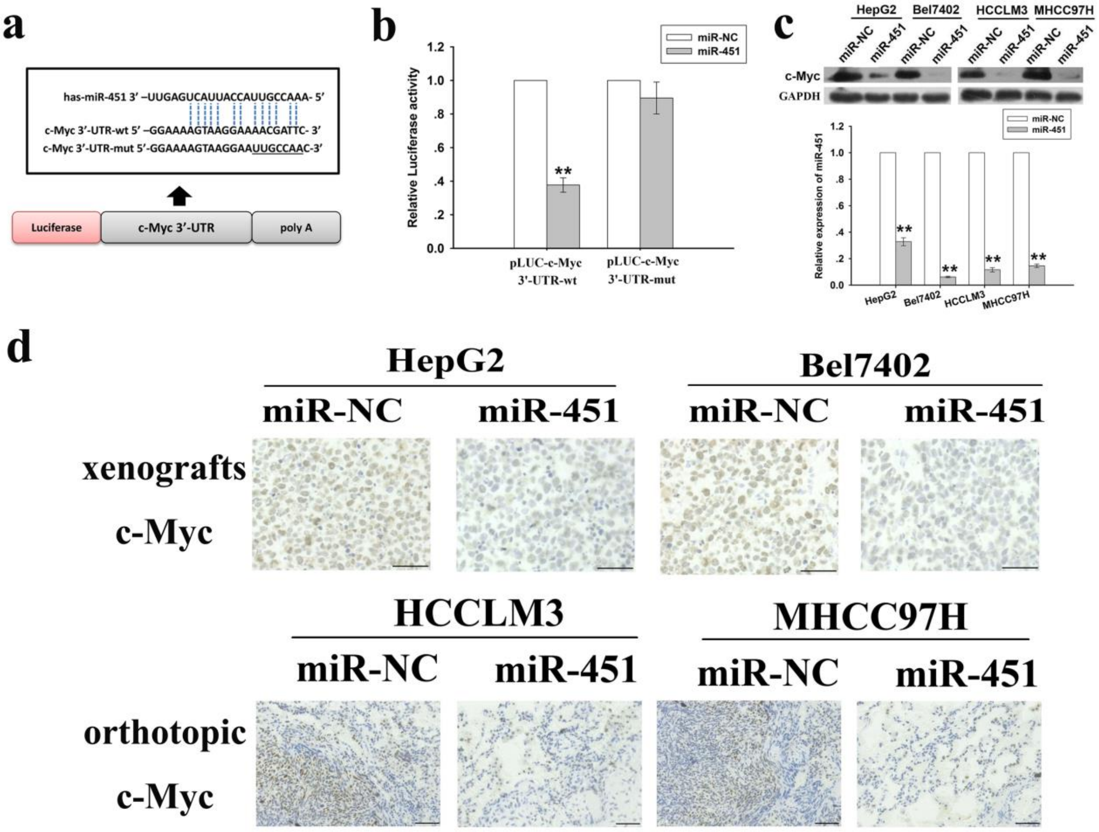

Supplementary Figure 4: MiR-451 directly target c-Myc in HCC. (a)The predicted human miR-451 target sequence in the wild-type or mutant c-Myc 3'-UTR fragment was cloned downstream of the luciferase reporter gene pLUC-Luv vector. (b)miR-451 induced a notable decrease in relative luciferase activity of c-Myc containing a wildtype 3'-UTR but not significantly suppress that of c-Myc with a mutant 3'-UTR after co-transfected plasmid with wild-type or mutant c-Myc 3'-UTR fragment and pcDNA/miR-NC or pcDNA/miR-451. The histogram of luciferase activity were normalized by mean±SD in three independent experiments. **P*<0.05, ***P*<0.01. (c) Western blot showed that upregulation of miR-451 result in a sharply drop of c-Myc expression in HCC cells. (d) In the both xenografts tumors and orthotopic lung implanted model tumors of miR-451 overexpressed HCC cells showed much weaker staining of c-Myc protein, compared with those of control groups.

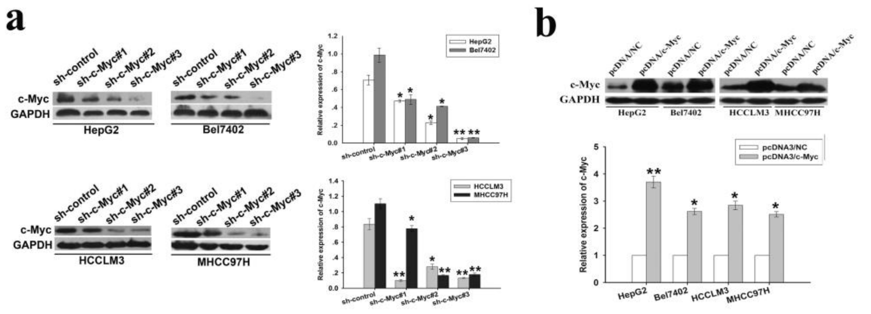

Supplementary Figure 5: The efficiency of transfected with c-Myc overexpressed plasmid and sh-c-Myc plasmid. (a) Expression level of c-Myc in HCC cells transfected with short hairpin RNA (shRNA) pSil/shc-Myc was detected via western blot. The results indicated that pSil/shc-Myc#3 held the best silencing efficiency on c-Myc in HCC cells. (b) Expression level of c-Myc in HCC cells transfected with pcDNA/c-Myc was detected via western blot. pcDNA/c-Myc was shown to mediate overexpression of c-Myc.

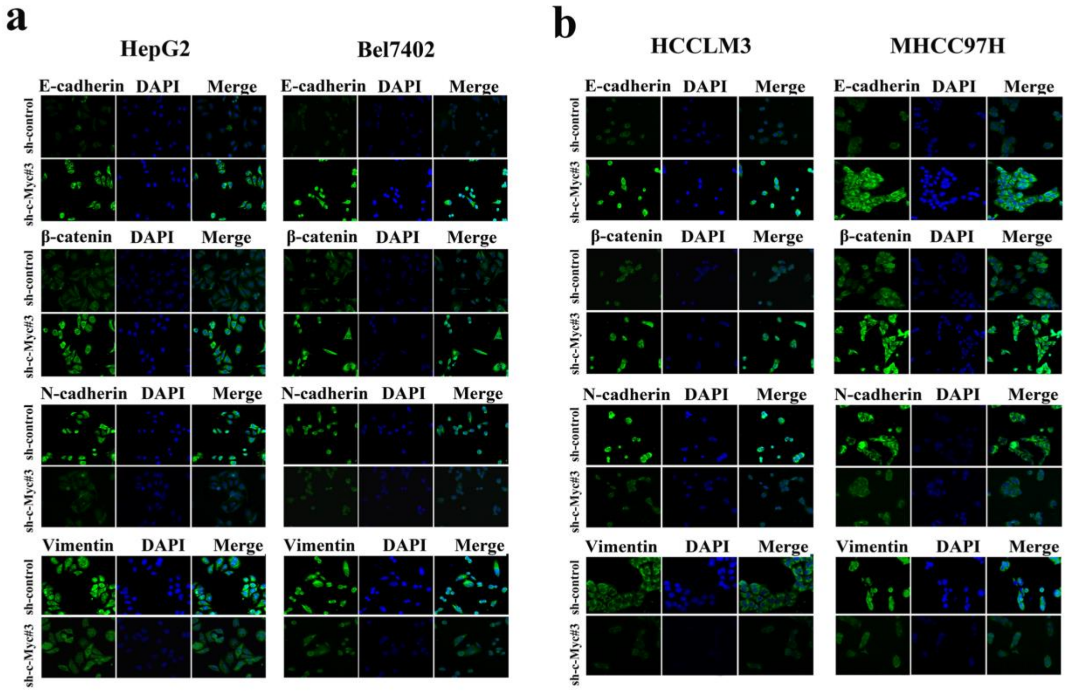

Supplementary Figure 6: Immunofluorecence assays were determine EMT phenotype of HCC cells influenced by c-Myc downregulation. (a)The expression of epithelial markers such as E-cadherin and β-catenin were remarkable increased in HCC cells transfected with pSil/shc-Myc#3, while the mesenchymal protein markers like N-cadherin and Vimentin were dramatically reduced in HepG2 and Bel7402. (b)Same results were shown in high initial metastatic HCC cells HCCLM3 and MHCC97H.

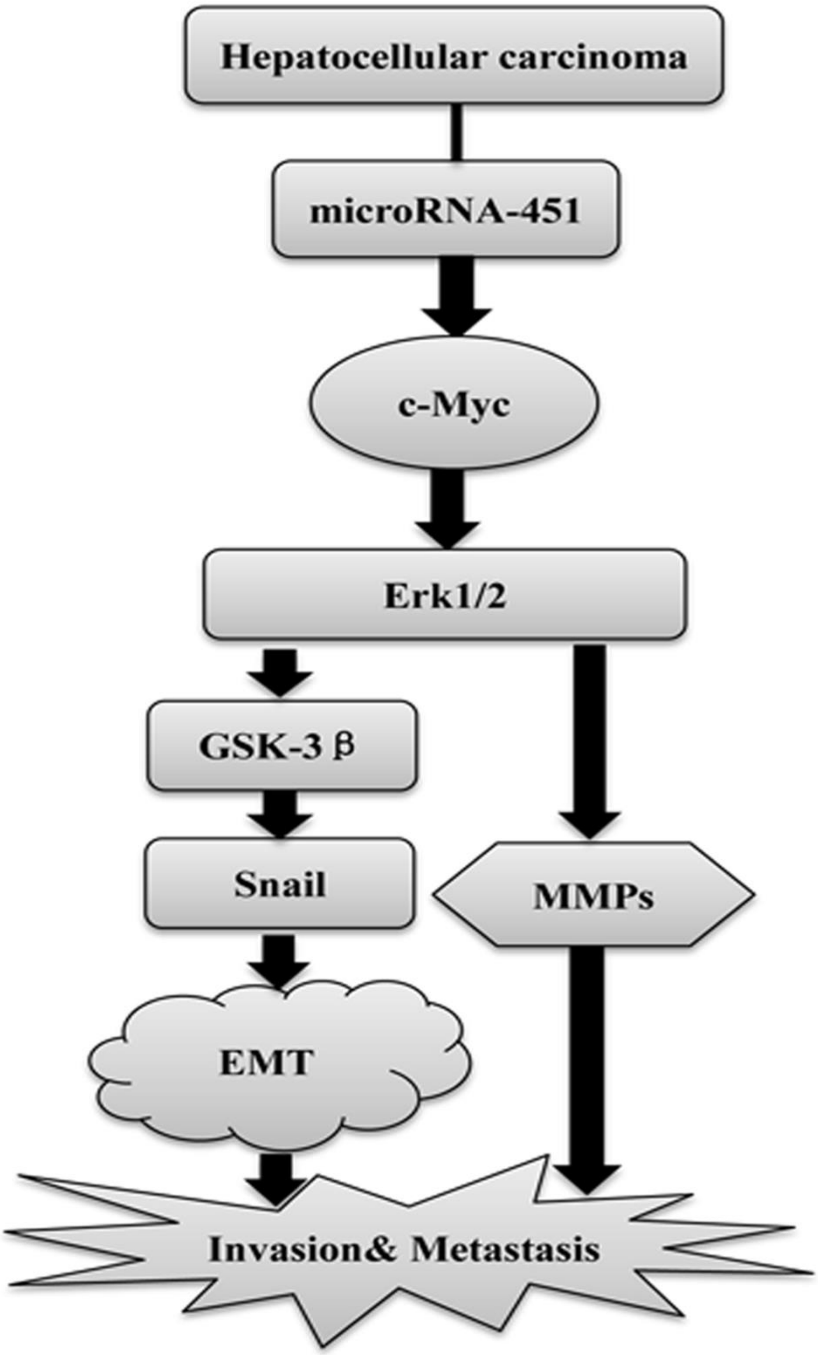

Supplementary Figure 7: The miR-451/c-Myc/Erk1/2/GSK-3β or MMPs signaling pathway might be involved in EMT and metastasis of HCC cells. Schematic diagram was illustrated that miR-451 could suppress tumor migration, invasion and metastasis via regulating EMT process and MMPs family expression. Proliferation was influenced by Cyclin D1, NF-κB and Bcl-2/Bax ratio as reported. The activation of miR-451/c-Myc/ERK axis is probably a significant mechanism in invasion and metastasis, it would be a critical therapeutic target in HCC.
